# Defining the healthy "core microbiome" of oral microbial communities

**DOI:** 10.1186/1471-2180-9-259

**Published:** 2009-12-15

**Authors:** Egija Zaura, Bart JF Keijser, Susan M Huse, Wim Crielaard

**Affiliations:** 1 Department of Cariology, Academic Centre for Dentistry Amsterdam (ACTA), University of Amsterdam and Free University Amsterdam, the Netherlands; 2 TNO Quality of Life, Business Unit Food and Biotechnology Innovations, Microbial Genomics Group, Zeist, the Netherlands; 3 Josephine Bay Paul Center for Comparative Molecular Biology and Evolution, Marine Biological Laboratory, Woods Hole, MA, USA

## Abstract

**Background:**

Most studies examining the commensal human oral microbiome are focused on disease or are limited in methodology. In order to diagnose and treat diseases at an early and reversible stage an in-depth definition of health is indispensible. The aim of this study therefore was to define the healthy oral microbiome using recent advances in sequencing technology (454 pyrosequencing).

**Results:**

We sampled and sequenced microbiomes from several intraoral niches (dental surfaces, cheek, hard palate, tongue and saliva) in three healthy individuals. Within an individual oral cavity, we found over 3600 unique sequences, over 500 different OTUs or "species-level" phylotypes (sequences that clustered at 3% genetic difference) and 88 - 104 higher taxa (genus or more inclusive taxon). The predominant taxa belonged to Firmicutes (genus *Streptococcus*, family *Veillonellaceae*, genus *Granulicatella*), Proteobacteria (genus *Neisseria*, *Haemophilus*), Actinobacteria (genus *Corynebacterium*, *Rothia*, *Actinomyces*), Bacteroidetes (genus *Prevotella*, *Capnocytophaga, Porphyromonas*) and Fusobacteria (genus *Fusobacterium*).

Each individual sample harboured on average 266 "species-level" phylotypes (SD 67; range 123 - 326) with cheek samples being the least diverse and the dental samples from approximal surfaces showing the highest diversity. Principal component analysis discriminated the profiles of the samples originating from shedding surfaces (mucosa of tongue, cheek and palate) from the samples that were obtained from solid surfaces (teeth).

There was a large overlap in the higher taxa, "species-level" phylotypes and unique sequences among the three microbiomes: 84% of the higher taxa, 75% of the OTUs and 65% of the unique sequences were present in at least two of the three microbiomes. The three individuals shared 1660 of 6315 unique sequences. These 1660 sequences (the "core microbiome") contributed 66% of the reads. The overlapping OTUs contributed to 94% of the reads, while nearly all reads (99.8%) belonged to the shared higher taxa.

**Conclusions:**

We obtained the first insight into the diversity and uniqueness of individual oral microbiomes at a resolution of next-generation sequencing. We showed that a major proportion of bacterial sequences of unrelated healthy individuals is identical, supporting the concept of a core microbiome at health.

## Background

The commensal human microbiome is estimated to outnumber the amount of human body cells by a factor of ten [1]. These complex microbial communities are normal residents of the skin, the oral cavity, vaginal and intestinal mucosa and carry a broad range of functions indispensable for the wellbeing of the host [2]. Usually we only become aware of their presence when the balance between the microbiota and the host is lost, and disease is manifest. This is reflected in the ample knowledge on the human microbiome at the state of disease as opposed to the limited picture we have of the healthy microbiome. In order to diagnose and treat disease at an early and reversible stage one needs to describe the commensal microbiome associated with health. For example, understanding changes in the oral microbiome at the early stages of periodontitis and dental caries, the most prevalent chronic oral diseases, would allow diagnosis and treatment before the appearance of periodontal pockets or dental hard tissue loss.

Recent advances in sequencing technology, such as 454 pyrosequencing provides hundreds of thousands of nucleotide sequences at a fraction of the cost of traditional methods [3]. This deep sequencing has revealed an unexpectedly high diversity of the human oral microbiome: dental plaque pooled from 98 healthy adults comprised about 10000 microbial phylotypes [4]. This is an order of magnitude higher than previously reported 700 oral microbial phylotypes as identified by cultivation or traditional cloning and sequencing [5]. Moreover, by pooling about 100 individual microbiomes and pyrosequencing these, the ecosystem still appeared undersampled: the ultimate diversity of the oral microbiome was estimated to be around 25000 phylotypes [4].

If "everything is everywhere, but, the environment selects" [6], then a healthy oral microbiome should be dominated by a "core microbiome" characteristic for health. These abundant phylotypes would maintain the functional stability and homeostasis necessary for a healthy ecosystem. To date though, there is no information available on how many of the 25000 phylotypes [4] actually contribute to a single oral cavity and how common or exclusive individual oral microbiomes of unrelated healthy individuals are.

The oral cavity differs from all other human microbial habitats by the simultaneous presence of two types of surfaces for microbial colonization: shedding (mucosa) and solid surfaces (teeth or dentures). This intrinsic property of the oral cavity provides immense possibilities for a diverse range of microbiota. Once the symbiotic balance between the host and the microbiota is lost, these microbiota may become involved in disease. For instance, the tongue, with its mucosal 'crypts' which allow anaerobic microbiota to flourish, is an established source of halitosis [7]. Approximal (adjoining) surfaces between adjacent teeth have limited access to fluorides and saliva, and therefore have a predilection for dental caries [8]. To gather as complete information as possible on the healthy oral microbiome, microbial samples should be obtained from various ecological niches throughout the oral cavity.

Here we present the first description of diversity, uniqueness and the level of overlap of microbiomes of three healthy individual oral cavities at various intraoral niches (different dental surfaces, cheek, hard palate, tongue and saliva) at the probing depth as provided by targeted pyrosequencing of the V5-V6 hypervariable region of the small subunit ribosomal RNA.

## Results and Discussion

### The overall sequence data

In total, 452071 reads passed the quality control filters. Recent publications [9,10] have identified the potential inflation of richness and diversity estimates caused by low-quality reads (pyrosequencing noise). Reads with multiple errors can form new OTUs if they are more distant from their real source than the clustering width. These reads are relatively rare and most commonly occur as singletons or doubletons. To preclude the inclusion of sequencing artifacts or potential contaminants from sample processing, and to avoid diversity overestimation, we included only sequences occurring at least five times in further analyses. By doing so, we have also removed many less frequent but valid sequences representing the rare members of the microbiome.

The final data contained 298261 reads and resulted in 6315 unique sequences (Table 1, Table 2). The average length of sequence reads was 241 nt. The stringent selection of sequences (the cut-off of 5 reads) and individual labelling and sequencing of 29 samples on a single pyrosequencing plate have largely reduced the depth of pyrosequencing resolution. On average, 10000 reads per sample were obtained instead of the 400000 reads possible when using a full plate for a single sample. Our findings on diversity, therefore, should be considered conservative.

**Table 1:** Participant details and number of sequences, OTUs and higher taxa.

Individual, Age	Birth Country	All Reads	Reads Analyzed^a^	Unique Sequences	OTUs at 3% Difference^**b**^	OTUs at 6% Difference^**b**^	OTUs at 10% Difference^**b**^	Higher Taxa^**c**^
S1, 39	The Netherlands	154530	100226	4124	630	418	269	95
S2, 29	Brazil	132649	86224	3668	541	370	237	88
S3, 45	The Netherlands	164892	111811	4293	649	434	282	104

^a ^Only reads that were observed five or more times were included in the analyses.

^b ^Sequences were clustered into Operational Taxonomic Units (OTUs) at 3%, 6% or 10% genetic difference.

^c ^Higher taxa refers to genus or to a more inclusive taxon (family, order, class) when sequence could not be confidently classified to the genus level.

**Table 2:** Distribution of reads, unique sequences, OTUs and shared microbiome (sequences and OTUs) per phylum.

Phylum	Number of Reads (% of all)^**a**^	Unique Sequences (% of all)^**a**^	Number of Shared Sequences^**b**^	% of Reads with Shared Sequences	Number of OTUs (% of all)^**c**^	Number of Shared OTUs^**d**^	% of Reads with Shared OTUs
Actinobacteria	73092 (25%)	1541 (24%)	520	20%	194 (24%)	94	24%
Bacteroidetes	32666 (11%)	748 (12%)	118	6%	132 (16%)	44	9%
Cyanobacteria	28 (0.01%)	4 (0.06%)	1	0.005%	3 (0.4%)	1	0.006%
Firmicutes	107711 (36%)	2283 (36%)	719	27%	230 (28%)	131	35%
Fusobacteria	14103 (5%)	233 (4%)	74	3%	37 (5%)	23	4%
Proteobacteria	65778 (22%)	1294 (20%)	212	12%	183 (22%)	77	20%
Spirochaetes	407 (0.1%)	18 (0.3%)	2	0.06%	8 (1%)	2	0.1%
TM7	3853 (1%)	127 (2%)	13	0.4%	14 (2%)	7	0.8%
Unclassified Bacteria	623 (0.2%)	67 (1%)	1	0.002%	17 (2%)	8	0.1%
Total	298261 (100%)	6315 (100%)	1660	66%	818 (100%)	387	93%

^a ^- sum of all samples of three individuals; only sequences that were present at least 5 times are included

^b ^- number of unique sequences that were common in all three microbiomes

^c ^- number of all OTUs (sequences that clustered at 3% genetic difference)

^d ^- number of OTUs (sequences clustered at 3% genetic difference) that were common in all three microbiomes

### Clustering of the overall data in phylotypes

Clustering the unique sequences into operational taxonomic units (OTUs) at a 3% genetic distance resulted in 818 different OTUs (Table 1, Additional file 1). A 97% identity in 16S rRNA gene sequences is commonly used to group "species-level" phylotypes [1,11,12]. A 3% variation within a short hypervariable region of the small subunit (SSU) rRNA gene may not correlate exactly with a 3% variation along the entire SSU rRNA gene. In fact, the correlation between genetic differences may well vary with different regions of the gene, and in different classes of organisms. However, most microbial diversity projects to date have used 3% OTUs [1,13,14], and to be consistent with other research using pyrosequencing sequences we have chosen to use 3% OTUs as well. We have also clustered sequences into OTUs using more conservative genetic differences of 6% and 10% (Table 1, Additional file 2, Additional file 3). In the further text however we refer only to OTUs at the 3% difference. These OTUs were grouped in 112 higher taxa (Additional file 4) consisting of 78 genera and 34 more inclusive taxa (*e.g*., family, order, class), representing eight bacterial phyla (Table 2).

**Figure 1:**
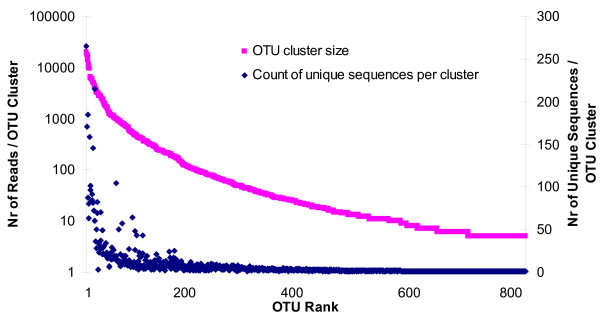
**The size of OTU clusters and the number of unique sequences per cluster**. The number of reads within each OTU (sequences that clustered at 3% genetic distance level) and the number of unique sequences per OTU are plotted in the rank order of OTU cluster size (high to low).

The size of the OTUs (number of reads per OTU) correlated significantly (p < 0.001; Spearman's rho 0.930) with the number of unique sequences within an OTU (Figure 1), *i.e*., the most abundant OTUs harboured the highest counts of unique sequences. An obvious outlier was one abundant OTU (0.9% of all reads), classified as *Fusobacterium *which contained only three unique sequences. Six other abundant OTUs (1.4 - 6.7% of all reads) contained more than 140 (range 145 - 265) unique sequences each. Four of these OTUs were assigned to the genus *Streptococcus *(OTU ID 803; 165; 230; 262), one to the genus *Corynebacterium *(ID 145), and one to the genus *Neisseria *(ID 637). Two-thirds of all OTUs contained a single sequence; however these were low abundance OTUs (5 - 49 reads), together contributing to just 0.7% of all reads (Figure 1, Additional file 1).

### Diversity and taxonomy of individual microbiomes

**Figure 2:**
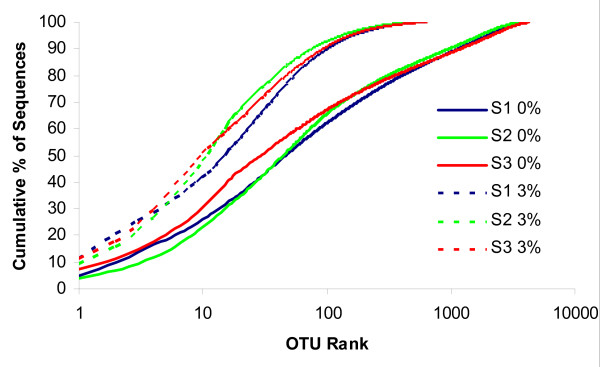
**The relative abundance of OTUs per individual**. Relative abundance of OTUs based on all unique sequences (0%, solid lines) and OTUs within genetic distances that do not exceed 3% difference (3%, dashed lines) per individual S1, S2 and S3, respectively. The x-axis indicates the individual OTUs, ranked according to their relative abundance (high to low). The y-axis indicates the cumulative abundance of the OTUs.

Within an individual oral cavity, over 3600 sequences comprising over 500 "species-level" phylotypes (Figure 2) and 88 - 104 higher taxa (genus level or above) were found (Table 1, Additional file 4). This richness is considerably higher than the 34 to 72 phylotypes and the 6 to 30 genera previously described using conventional cloning and sequencing [15,16]. The predominant taxa belonged to Firmicutes (genus *Streptococcus*, family *Veillonellaceae*, genus *Granulicatella*), Proteobacteria (genus *Neisseria*, *Haemophilus*), Actinobacteria (genus *Corynebacterium*, *Rothia*, *Actinomyces*), Bacteroidetes (genus *Prevotella*, *Capnocytophaga, Porphyromonas*) and Fusobacteria (genus *Fusobacterium*) (Additional file 4).

About 100 "species-level" phylotypes (118, 97 and 112 phylotypes in the microbiome of individual S1, S2 and S3, respectively) belonged to abundant OTUs of the individual microbiome (Additional file 1). A phylotype was considered abundant if it contributed to at least 0.1% of the microbiome. These abundant phylotypes together contributed to 92 - 93% of each microbiome.

As with a pooled oral microbiome [4] and individually sequenced gut microbiomes [13], each individual oral microbiome in this study was dominated by a few sequences while most sequences were rare and contributed to the "long tail" effect (Figure 2).

### Overlap of three individual oral microbiomes

#### Unique sequences

**Figure 3:**
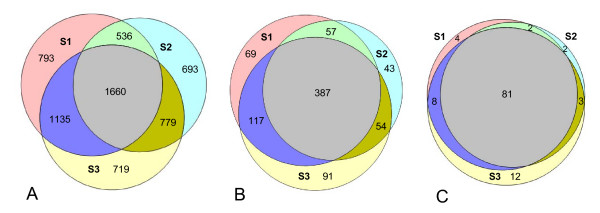
**The extent of overlap of oral microbiome between three individuals**. The extent of overlap between subjects S1 (pink circle), S2 (light blue circle) and S3 (yellow circle) at the level of A) unique sequences, B) OTUs clustered at 3% difference and C) higher taxa (genus or more inclusive taxon). The data was obtained by combining all samples of the respective individual microbiome. The Venn Diagrams show that 26% of the unique sequences, 47% of the OTUs and 72% of the higher taxa were common (area in grey) to the three individuals.

Twenty-six percent (1660 sequences) of the unique sequences were found in all three microbiomes and 65% in at least two microbiomes (Figure 3A). Of all reads, 66% belonged to sequences that were shared by three microbiomes (Table 2). Nine sequences were highly abundant (0.5 - 5.8% of the reads) across all individuals: they contributed to 11%, 9% and 21% of the microbiome of individuals S1, S2 and S3, respectively (the full list of the taxonomy and abundance of the overlapping sequences is given in Additional file 5). Two of these sequences were assigned to the genus *Streptococcus*, two to the family *Veillonellaceae*, one each to the genera *Granulicatella *(Firmicutes), *Corynebacterium*, *Rothia *(Actinobacteria), *Porphyromonas *(Bacteroidetes) and *Fusobacterium *(Fusobacteria).

On the other hand, 17-19% of the unique sequences originating from a single oral cavity were not shared with either of the other two microbiomes (Table 3). Combined, these "exclusive" sequences contributed to 11 - 20% of the total count of reads within an individual microbiome. Within an individual, one to six "exclusive" sequences were highly abundant (Table 3). Sequencing of a larger number of individual microbiomes is necessary for assessing the true exclusivity of these abundant individual-specific sequences.

**Table 3:** Relative abundance of individual-specific ("exclusive") sequences

Individual	% Sequences "Exclusive"	% of Reads with "Exclusive" Sequences	Taxonomy of Predominant "Exclusive" Sequences^**a**^	% of Reads	Nr of Samples^**b**^
S1	19	20	Firmicutes;Bacilli;*Lactobacillales;Streptococcaceae;Streptococcus*	4.4	3
			Bacteria;Bacteroidetes;*Bacteroidia;Bacteroidales*	1.2	9
			Bacteria;Proteobacteria;Gammaproteobacteria;*Pasteurellales;Pasteurellaceae*	1.2	8
			Bacteria;Proteobacteria;Gammaproteobacteria;*Pasteurellales;Pasteurellaceae;Haemophilus*	0.6	4
			Bacteria;Proteobacteria;Gammaproteobacteria;*Pasteurellales;Pasteurellaceae*	0.6	5
			Bacteria;Proteobacteria;Gammaproteobacteria;*Cardiobacteriales;Cardiobacteriaceae;Cardiobacterium*	0.5	4

S2	19	12	Bacteria;Proteobacteria;Betaproteobacteria;*Neisseriales;Neisseriaceae;Neisseria*	0.6	3

S3	17	11	Bacteria;TM7	0.7	3
			Bacteria;Firmicutes;Bacilli;*Bacillales;Staphylococcaceae;Gemella*	0.5	7
			Bacteria;Actinobacteria;Actinobacteria;*Actinomycetales;Corynebacteriaceae;Corynebacterium*	0.5	5

^a ^- sequence was considered predominant if it contributed to at least 0.5% of the individual microbiome

^b ^- number of samples of the particular individual where the respective "exclusive" sequence was found

#### Phylotypes

**Figure 4:**
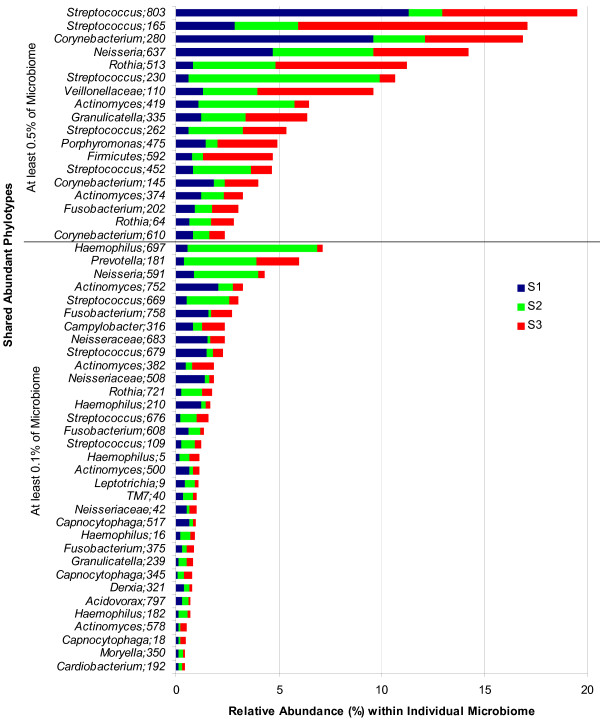
**Shared abundant phylotypes in three oral microbiomes and their relative abundance**. Relative abundance of shared phylotypes within an individual microbiome. Only abundant phylotypes that contributed to at least 0.1% of the individual microbiome are shown. The most abundant phylotypes (≥0.5% of the microbiome) are grouped separately in the upper panel. Phylotypes were defined as OTUs clustering sequences at a 3% genetic difference. The highest taxon (in most cases, genus) at which the OTU was identified, is shown together with the cluster identification number. The full list of OTUs is available in Additional file 1. Different colours indicate three different microbiomes, S1, S2 and S3, respectively.

All three microbiomes shared 387 (47%) of 818 OTUs (Figure 3B). These overlapping phylotypes together contributed to 90 - 93% of each microbiome (Additional file 1). Fifty-one of these shared OTUs were abundant (≥0.1% of microbiome) and together occupied 62 - 73% of the individual microbiome (Figure 4).

Sixty-nine, 43 and 91 OTUs originated from one particular microbiome and contributed to 3.9%, 0.5% and 0.9% of the microbiome from individual S1, S2 and S3, respectively. Interestingly, all unique OTUs from either S2 or S3 were present at low abundance, while in S1 four of 69 unique phylotypes were relatively abundant (≥ 0.1% of the microbiome). One phylotype (OTU ID 774, *Pasteurellaceae*) contributed to 2.2% of this microbiome and was preferentially found around the molar tooth (buccal, lingual and approximal surfaces of tooth 16) and in the sample obtained at the hard palate.

The OTUs representing different phyla were not equally shared among the individuals (Table 2). The lowest similarity was observed in Spirochaetes (25% common OTUs), followed by Bacteroidetes and Cyanobacteria (33%), Proteobacteria (42%), Actinobacteria (48%), candidate division TM7 (50%), Firmicutes (57%), while the highest similarity was found in Fusobacteria (62%). The low similarity among the OTUs of Spirochaetes among the three microbiomes could be due to low abundance of this phylum in the different samples. Since a high prevalence of Spirochaetes in dental plaque is associated with periodontal disease [17], it would be interesting to assess the degree of similarity and diversity of these phylotypes in a group of periodontitis patients.

#### Higher taxa

At the higher taxonomic levels, 72% of all taxa (genus level or above) were shared by the three microbiomes, contributing to 99.8% of all reads. Only 2-11% of higher taxa were individual-specific (Figure 3C, Additional file 4). However, these taxa were found at a very low abundance (5-49 reads) and most likely were not a part of the commensal oral flora, and should be regarded as "transients".

The observed overlap in taxa and in phylotypes is unexpectedly high and considerably higher than the recently reported average of 13% similarity in phylotypes between any two hands from unrelated individuals [12]. Of even greater contrast to our findings are the comparisons of gut microbiomes which show no overlap in microbiota in unrelated individuals [1]. Instead of a core microbiome at an organismal lineage level, gut microbiomes harboured distinct core genes [1]. The most probable explanation in the observed exclusiveness of gut microbiomes is the close interplay of intestinal microbiota with the host.

In the abovementioned study on hand surface microbiomes, only five phylotypes were shared across the 102 hands sampled [12]. Human palms are continuously exposed to diverse biological and abiotic surfaces that may function as a microbial source, and furthermore, hands are regularly washed, allowing new communities of different origins to establish. This may explain the high diversity and relatively low overlap in hand palm communities. The situation is cardinally different in the oral cavity. Even though dental hygiene procedures (toothbrushing, flossing) effectively removes dental plaque, newly cleaned surfaces are continuously bathed in saliva. Saliva functions here as a transport medium for microorganisms from sites that were not affected by cleansing (tongue and other mucosal sites, gingival crevices, anatomical irregularities on tooth surfaces etc). Furthermore, the human mouth is a relatively stable ecosystem regarding temperature and saliva as a nutrient source. The contact of the oral cavity with external microbial sources is highest in the first years of human life [18], and is mostly limited to microorganisms in food or drinking water at a later age.

### Sample-specific profiles within individual oral microbiomes

**Figure 5:**
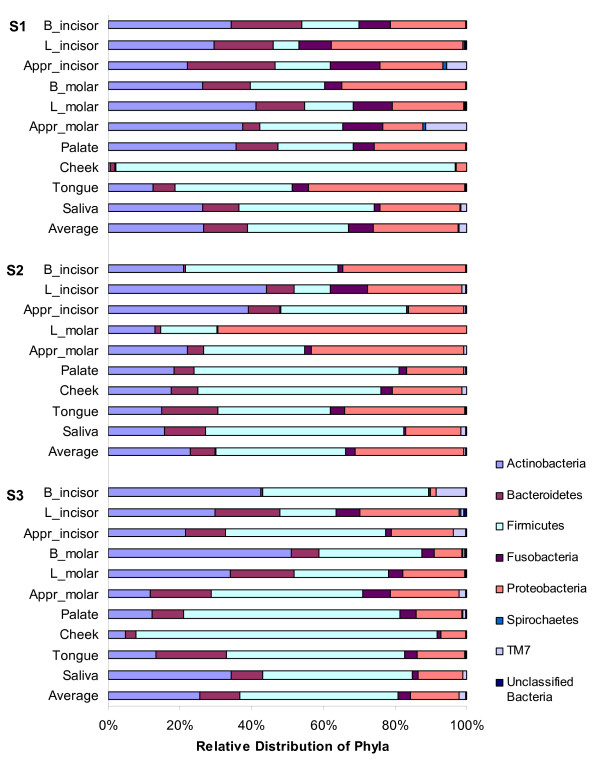
**Average and site-specific relative distribution of bacterial phyla in three individuals**. Average and site-specific relative distribution of bacterial phyla in three individuals (S1, S2 and S3). Unclassified bacteria were reads without a recognizable match in the full 16S rRNA reference database. Sample legend: B - buccal, L - lingual, Appr - approximal surface of either an incisor (a front tooth) or a molar tooth.

Even at the phylum level, distinct differences among various intraoral sites were observed, *e.g*. Firmicutes dominated the cheek mucosa of volunteers S1 and S3, while the relatively minor phylum, candidate division TM7, was overrepresented at the approximal sites of volunteer S1 and on incisor buccal and incisor approximal surfaces of volunteer S3 (Figure 5).

Fifteen taxa were found at all sites in all three individuals: the genera *Streptococcus, Neisseria, Corynebacterium, Rothia, Actinomyces, Haemophilus, Prevotella, Fusobacterium, Granulicatella, Capnocytophaga*, representatives of the *Veillonellaceae*, *Neisseriaceae *and *Pasteurellaceae *families, the *Bacteroidales *order and unclassified Firmicutes. Unclassified Bacteria and an additional four taxa were found in all but one sample: genus *Porphyromonas, Leptotrichia*, TM7 genera *incertae sedis *and *Campylobacter *(Additional file 6).

As mentioned above (Figure 2), a few sequences dominated each individual microbiome. Three of the sequences were found across all 29 samples that originated from three individuals: two *Veillonellaceae *family members (phylum Firmicutes) and one *Fusobacterium *genus member (phylum Fusobacteria). This latter ubiquitous sequence accounted for 34% of *Fusobacterium *reads and for 1% of the total reads (Additional file 5). The latter finding is especially interesting in the light of the central role fusobacteria play in mediating coaggregation of non-aggregating microbiota and their importance as a structural component of both healthy and disease-associated dental plaque [19].

Within an individual oral cavity, 36 - 51% of the unique sequences were found solely in a single sample and mostly at a low abundance. About 600-750 sequences per individual were found only once. Among these, numerous representatives of commensal oral microorganisms, as well as non-commensal microbiota, such as *Vibrio*, *Salinivibrio *and other Gammaproteobacteria were present. Even though these sequences were found as singletons in a particular microbiome, they had to be present at least five times across all three microbiomes according to the cut-off we applied.

Not all sequences that were found at a single site were rare: 16 of the sample-specific sequences (ten, two and four sequences in individuals S1, S2 and S3, respectively) were found at least 100 times (maximum 321 times) in a particular sample (data not shown). Surprisingly, all four abundant sample-specific sequences from volunteer S3 (two streptococci, *Granulicatella *and *Corynebacterium*) and five of the ten abundant sample-specific sequences from volunteer S1 (three streptococci, *Haemophilus *and *Acidovorax*) were found solely in the saliva sample of the respective individuals. The relatively high abundance of these saliva-specific organisms suggests that they are a part of the commensal oral microbiota. The most likely source of these organisms is a niche that was not specifically sampled but was exposed to saliva, *e.g*., tonsils, back of the tongue or subgingival plaque. Tonsils, for instance, have been shown to harbour a more diverse community than intraoral mucosal or dental sites [15].

**Figure 6:**
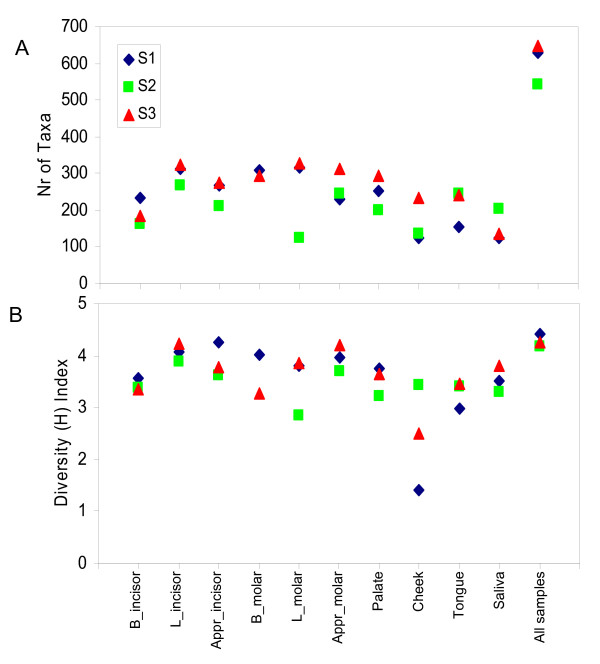
**Diversity statistics of individual samples**. Diversity statistics: A) number of taxa (OTUs clustering sequences at a 3% genetic difference) per sampling site for each individual; B) diversity index - Shannon diversity index, H, taking into account the number and the proportion (abundance) of taxa.

On average, each individual sample harboured 266 "species-level" phylotypes (SD 67; range 123 - 326) (Figure 6A). This is again considerably higher than the previously reported 4 - 28 species per site using traditional cloning and sequencing methods [15] or 10 - 81 species using a 16S rRNA gene-based microarray [20].

A trend for a higher diversity was observed in the samples taken at the approximal surfaces and the lingual surface of the front teeth (Figure 6B). The approximal surfaces, also known as plaque stagnations sites, are protected from regular toothbrushing. Although volunteers were asked to brush their teeth 12 hr before the samples were collected, the use of interdental oral hygiene means such as floss or toothpicks was not controlled. It is likely that older and thus more diverse plaque [21] was sampled at these sites. Higher diversity of the plaque from the lingual surface of the front tooth but not that of the molar tooth suggests that the composition of plaque of the lingual surface of the front tooth might be influenced by the anatomy of this surface - a protruding rounded tubercle at the gingival third of the crown, near the gingival sulcus. The area near the sulcus, protected by the tubercle, may have provided a niche suitable for more diverse microorganisms than anatomically flat lingual surface of the molar.

The two cheek samples from individual S1 and individual S3 showed the lowest diversity among all samples (Figure 6B). These samples were dominated by only two OTUs each, identified as streptococci, with 70 sequences comprising 13% of all reads in the sample from S1, and 46 sequences comprising 17% of the reads in the cheek sample from S3. The closest match to these OTUs was *Streptococcus mitis *which is known to produce immunoglobulin A1 protease. This enzyme is important for the ability of bacteria to colonize mucosal membranes in the presence of S-IgA antibodies in saliva [22] and might explain high dominance of these phylotypes in these particular samples. Notably, the cheek sample from S3 still contained one of the highest counts of taxa (234 phylotypes), but obviously at a very low abundance.

**Figure 7:**
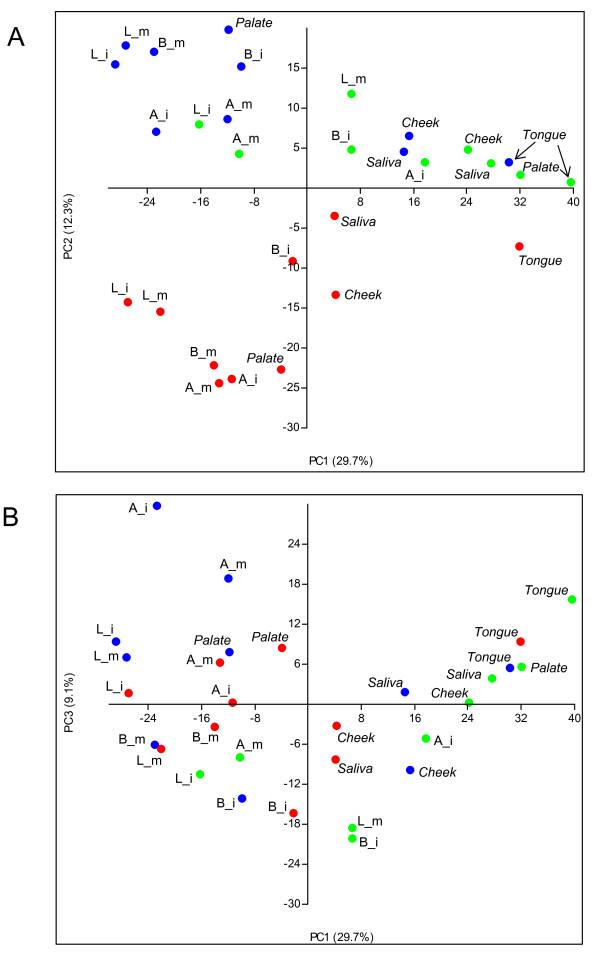
**Principal Component Analysis results on individual samples**. Principal Component Analysis (PCA) results on all individual samples at the level of OTUs clustering sequences at a 3% difference: A) the plot of the PCA axis 1 (accounting for 29.7% of intersample variation) and the axis 2 (12.3% of intersample variation); B) the plot of the PCA axis 1 and the axis 3 (9.1% of intersample variation). Blue dots - samples from individual S1, green dots - samples from individual S2, red dots - individual S3. A - approximal, B - buccal, L - lingual surface of i - incisor or m - molar tooth, respectively. Data were normalized to an equal number of reads per sample and log2 transformed.

Dimensional reduction of the OTU data by principal component analysis (PCA) explained 51% of the total variance among the individual samples by the first three components (Figure 7A-B; PCA loadings and respective taxa are listed in Additional file 7). The greatest component (PC1, 29.7% of variance) discriminated between the samples of dental and mucosal origin, especially in individuals S1 and S3. The second greatest component (PC2, 12.3% of variance) discriminated all samples of volunteer S3 from the samples of S1 and S2. The third component (PC3, 9.1% of variance) increased the separation of the samples of mucosal and dental origin, *e.g*. all three tongue samples aligning in the vicinity of each other (Figure 7B), supporting the earlier findings that the tongue has a specific microbial profile [20]. Since saliva is easily and non-invasively accessible it is a popular sample in oral epidemiology and microbiome diversity [4,16] studies. In our study, the profiles of the saliva samples were closer to communities obtained from mucosal than dental sites, which is in line with the results of a large scale survey on 225 healthy subjects where 40 selected bacterial species were followed using DNA-DNA hybridization technique [23].

In order to explore if the location in the oral cavity has an effect on the microbiota of the particular niche (lingual, buccal or approximal surface of the tooth), we sampled two distant teeth - the front tooth and the first molar. No pattern could be found among the samples from individual S2. However, both distantly situated lingual samples from individual S1 and S3, as well as both approximal samples from individual S3, showed higher similarity than the buccal samples of the respective individual (Figure 7A-B). The differences in the intraoral conditions such as salivary flow, lip or cheek movement, chewing forces and food clearance, may have had a higher impact on buccal than lingual or approximal surfaces of the two regions of the oral cavity.

## Conclusions

The major proportion of oral microbiomes was common across three unrelated healthy individuals, supporting the concept of a core-microbiome at health. The site specificity of the oral microbiome, especially between mucosal and dental sites and between saliva and dental sites, should be considered in future study designs. Sequencing large sub-populations in longitudinal clinical trials at defined intermediate stages from health to disease will provide oral health professionals with valuable information for future diagnostic and treatment modalities.

## Methods

### Samples

Three healthy Caucasian male adults (Table 1) with no antibiotic use in the past three months participated in the study after signed informed consent. The study was approved by the Medical Ethical Committee of the Free University Amsterdam. Each individual had a full set of natural dentition and none of them wore any removable or fixed prosthetic appliances, they had no clinical signs of oral mucosal disease and did not suffer from halitosis, did not have caries (white spot lesions of enamel or dentin lesions) or periodontal disease. The periodontal health was defined as no periodontal pockets deeper than 3 mm and no bleeding on probing at more than 10% of gingival sites. The sites that were sampled did not show any bleeding. In selecting healthy volunteers for experimental gingivitis studies, gingiva is considered healthy if bleeding on marginal probing is present at less than 20-25% of gingival sites [24,25].

Samples were collected in the morning, 12 hr after tooth brushing and 2 hr after the last food and/or drink intake. Parafilm-chewing stimulated saliva was collected and mixed 1:2 with RNAProtect (Qiagen, Hilden, Germany). For supragingival plaque sampling, three intact dental surfaces around a single upper incisor (tooth 11 buccally, lingually, and approximal surfaces of teeth 11/12) and around an upper molar (tooth 16 buccally, lingually, and approximal surfaces of teeth 15/16) were selected. Mucosal swabs were collected from the cheek, hard palate and tongue surface. The mucosal and dental surface swabs were collected using a sterile microbrush (Microbrush International, Grafton, USA). To sample buccal and lingual dental surfaces, the microbrush was moved over the enamel from mesial to distal curvature of the tooth crown along the gingival margin and tooth-surface border. The cheek mucosa and hard palate were sampled by making a circular motion of the microbrush over the central part of cheek mucosa or hard palate while applying slight pressure. The tongue swab was collected by several strokes over the first two thirds of the tongue dorsum in anterior-posterior direction. After the sample was taken, the tip of the microbrush was placed into an Eppendorf vial with 0.2 ml RNAProtect solution and clipped off. Interproximal plaque from the approximal surfaces (11/12 and 15/16) was collected with unwaxed dental floss (Johnson & Johnson, Almere, the Netherlands). A piece of floss was carefully slid over the contact point and moved slowly upwards along both neighbouring approximal surfaces. Then one end of the floss was released and the floss was slowly pulled through the interdental space avoiding the contact with gingiva. Plaque was removed from the dental floss by drawing it through a slit cut in the lid of a Eppendorf vial [26] containing 0.2 ml RNAProtect solution. One sample (buccal molar surface) from individual S2 was lost in sample processing. All samples were stored at -80°C until further processing for DNA extraction.

### Molecular techniques

A 0.35-ml quantity of lysis buffer (AGOWA mag Mini DNA Isolation Kit, AGOWA, Berlin, Germany) was added to plaque and mucosal swab samples. A 0.1-ml quantity of saliva sample was transferred to a sterile screw-cap Eppendorf tube with 0.25 ml of lysis buffer. Then 0.3 g zirconium beads (diameter, 0.1 mm; Biospec Products, Bartlesville, OK, USA) and 0.2 ml phenol were added to each sample. The samples were homogenized with a Mini-beadbeater (Biospec Products) for 2 min. DNA was extracted with the AGOWA mag Mini DNA Isolation Kit (AGOWA, Berlin, Germany) and quantified (Nanodrop ND-1000; NanoDrop Technologies, Montchanin, DE, USA).

PCR amplicon libraries of the small subunit ribosomal RNA gene V5-V6 hypervariable region were generated for the individual samples. PCR was performed using the forward primer 785F (GGATTAGATACCCBRGTAGTC) and the reverse primer 1061R (TCACGRCACGAGCTGACGAC). The primers included the 454 Life Sciences (Branford, CT, USA) Adapter A (for forward primers) and B (for reverse primers) fused to the 5' end of the 16S rRNA bacterial primer sequence and a unique trinucleotide sample identification key.

The amplification mix contained 2 units of Goldstar DNA polymerase (Eurogentec, Liège, Belgium), 1 unit of Goldstar polymerase buffer (Eurogentec), 2.5 mM MgCl_2_, 200 μM dNTP PurePeak DNA polymerase Mix (Pierce Nucleic Acid Technologies, Milwaukee, WI), 1.5 mM MgSO_4 _and 0.2 μM of each primer. After denaturation (94°C; 2 min), 30 cycles were performed that consisted of denaturation (94°C; 30 sec), annealing (50°C; 40 sec), and extension (72°C; 80 sec). DNA was isolated by means of the MinElute kit (Qiagen, Hilden, Germany). The quality and the size of the amplicons were analyzed on the Agilent 2100 Bioanalyser with the DNA 1000 Chip kit (Agilent Technologies, Santa Clara, CA, USA) and quantified using Nanodrop ND-1000 spectrophotometer. The amplicon libraries were pooled in equimolar amounts in two separate pools. Each pool was sequenced unidirectionally in the reverse direction (B-adaptor) by means of the Genome Sequencer FLX (GS-FLX) system (Roche, Basel, Switzerland). Sequences are available at the Short Read Archive of the National Center for Biotechnology Information (NCBI) [NCBI SRA: SRP000913].

### Data analysis

GS-FLX sequencing data were processed as previously described [14]. In brief, we trimmed sequences by removing primer sequences and low-quality data, sequences that did not have an exact match to the reverse primer, that had an ambiguous base call (N) in the sequence, or that were shorter than 50 nt after trimming. We then used the GAST algorithm [27] to calculate the percent difference between each unique sequence and its closest match in a database of 69816 unique eubacterial and 2779 unique archaeal V5-V6 sequences, representing 323499 SSU rRNA sequences from the SILVA database [28]. Taxa were assigned to each full-length reference sequence using several sources including Entrez Genome entries, cultured strain identities, SILVA, and the Ribosomal Database Project Classifier [29]. In cases where reads were equidistant to multiple V5-V6 reference sequences, and/or where identical V5-V6 sequences were derived from longer sequences mapping to different taxa, reads were assigned to the lowest common taxon of at least two-thirds of the sequences. The operational taxonomic units (OTUs) were created by aligning unique sequences and calculating distance matrices as previously described [14] and using DOTUR [30] to create clusters at the 0.03, 0.06 and 0.1 level.

Only sequences that were found at least 5 times were included in the analyses. This strict and conservative approach was chosen to preclude inclusion of sequences from potential contamination or sequencing artefacts. To compare the relative abundance of OTUs among samples, the data were normalized for number of sequenced reads obtained for each sample. To reduce the influence of abundant taxa on principal component analyses, the normalized abundance data were log2 transformed. Shannon Diversity Index (H' = -Σ *p*_*i*_ln(*p*_*i*_) where *p*_*i *_is the proportion of taxon *i*) and Principal component analysis (PCA) were performed in PAST v. 1.89 [31]. The Venn diagrams were made with Venn Diagram Plotter v. 1.3.3250.34910 (Pacific Northwest National Laboratory http://www.pnl.gov/; http://omics.pnl.gov/. Spearman correlation between the size of OTUs and the number of unique sequences within each OTU was calculated using SPSS (Version14.0).

## Authors' contributions

EZ and WC have contributed to the design of the clinical study; EZ carried out clinical procedures; BJFK processed the samples; SMH performed sequence analyses; EZ, BJFK, SMH and WC drafted the manuscript. All authors read and approved the final manuscript.

## Supplementary Material

Additional file 1 **Full list and taxonomy of OTUs clustered at 3% difference in descending order of their relative abundance (%)**. This is an Excel file listing all 818 OTUs, number of unique sequences within each OTU, abundance and the taxonomic assignment of each OTU per individual S1, S2 and S3. Click here for file

Additional file 2 **Full list and taxonomy of OTUs clustered at 6% difference in descending order of their relative abundance (%)**. This is an Excel file listing all 517 OTUs, abundance and the taxonomic assignment of each OTU per individual S1, S2 and S3. Click here for file

Additional file 3 **Full list and taxonomy of OTUs clustered at 10% difference in descending order of their relative abundance (%)**. This is an Excel file listing all 320 OTUs, abundance and the taxonomic assignment of each OTU per individual S1, S2 and S3. Click here for file

Additional file 4 **Full list and relative abundance of higher taxa per individual microbiome**. This is an Excel file listing all 112 higher taxa (genera or more inclusive taxa when sequences could not be confidently classified to the genus level) and their relative abundance in oral microbiomes of three individuals: S1, S2 and S3. Click here for file

Additional file 5 **Relative abundance of 1660 unique sequences that were shared by three individuals (S1, S2 and S3)**. This Excel file lists the taxonomy of the sequences shared by three individuals, ranked by the abundance of these sequences in the total data set. The sequences are available at the Short Read Archive of NCBI as SRP000913. Click here for file

Additional file 6 **Full list and absolute abundance of higher taxa per individual sampling site**. This is an Excel file listing all 112 higher taxa (genera or more inclusive taxa when sequences could not be confidently classified to the genus level) and their abundance in 29 samples from three individuals: S1, S2 and S3. Data were not normalized. Click here for file

Additional file 7 **Full list of taxa and PCA loadings**. This is an Excel file listing the loadings of the first three components of the Principal Component Analysis (PCA) on all 818 OTUs (3% genetic difference) and all 29 samples (the corresponding PCA plots are shown in Figure 7). The loadings marked in bold and highlighted are above the arbitrary significance threshold of 1 or -1. The positive values are highlighted yellow; the negative values are highlighted turquoise. Click here for file

## References

[B1] Turnbaugh PJ Hamady M Yatsunenko T Cantarel BL Duncan A Ley RE Sogin ML Jones WJ Roe BA Affourtit JP Egholm M Henrissat B Heath AC Knight R Gordon JI A core gut microbiome in obese and lean twins Nature 2009 457 480 484 10.1038/nature07540 19043404 PMC2677729

[B2] Wilson M Bacteriology of Humans: An Ecological Perspective 2008 Malden, MA: Blackwell Publishing Ltd

[B3] Voelkerding KV Dames SA Durtschi JD Next-generation sequencing: from basic research to diagnostics Clin Chem 2009 55 641 658 10.1373/clinchem.2008.112789 19246620

[B4] Keijser BJF Zaura E Huse SM van der Vossen JMBM Schuren FHJ Montijn RC ten Cate JM Crielaard W Pyrosequencing analysis of the oral microflora of healthy adults J Dent Res 2008 87 1016 1020 10.1177/154405910808701104 18946007

[B5] Paster BJ Olsen I Aas JA Dewhirst FE The breadth of bacterial diversity in the human periodontal pocket and other oral sites Periodontol 2000 2006 42 80 87 10.1111/j.1600-0757.2006.00174.x 16930307

[B6] Baas-Becking LGM Geobiologie of Inleiding tot de Milieukunde 1934 The Hague: Van Stokkun & Zoon

[B7] Scully C Greenman J Halitosis (breath odor) Periodontol 2000 2008 48 66 75 10.1111/j.1600-0757.2008.00266.x 18715357

[B8] Zaura E Plaque stagnation sites and dental caries: Studies on dental biofilm and dentin demineralization in narrow grooves PhD thesis 2002 Amsterdam: Faculteit der Tandheelkunde, University of Amsterdam

[B9] Quince C Lanzen A Curtis TP Davenport RJ Hall N Head IM Read LF Sloan WT Accurate determination of microbial diversity from 454 pyrosequencing data Nat Meth 2009 6 639 641 10.1038/nmeth.1361 19668203

[B10] Kunin V Engelbrektson A Ochman H Hugenholtz P Wrinkles in the rare biosphere: pyrosequencing errors can lead to artificial inflation of diversity estimates Environ Microbiol in press 19725865 10.1111/j.1462-2920.2009.02051.x

[B11] Acinas SG Klepac-Ceraj V Hunt DE Pharino C Ceraj I Distel DL Polz MF Fine-scale phylogenetic architecture of a complex bacterial community Nature 2004 430 551 10.1038/nature02649 15282603

[B12] Fierer N Hamady M Lauber CL Knight R The influence of sex, handedness, and washing on the diversity of hand surface bacteria Proc Natl Acad Sci USA 2008 105 17994 17999 10.1073/pnas.0807920105 19004758 PMC2584711

[B13] Dethlefsen L Huse S Sogin ML Relman DA The pervasive effects of an antibiotic on the human gut microbiota, as revealed by deep 16S rRNA sequencing PLoS Biol 2008 6 e280 10.1371/journal.pbio.0060280 19018661 PMC2586385

[B14] Sogin ML Morrison HG Huber JA Mark Welch D Huse SM Neal PR Arrieta JM Herndl GJ Microbial diversity in the deep sea and the underexplored "rare biosphere" Proc Natl Acad Sci USA 2006 103 12115 12120 10.1073/pnas.0605127103 16880384 PMC1524930

[B15] Aas JA Paster BJ Stokes LN Olsen I Dewhirst FE Defining the normal bacterial flora of the oral cavity J Clin Microbiol 2005 43 5721 5732 10.1128/JCM.43.11.5721-5732.2005 16272510 PMC1287824

[B16] Nasidze I Li J Quinque D Tang K Stoneking M Global diversity in the human salivary microbiome Genome Res 2009 19 636 643 10.1101/gr.084616.108 19251737 PMC2665782

[B17] Ellen RP Galimanas VB Spirochetes at the forefront of periodontal infections Periodontol 2000 2005 38 13 32 10.1111/j.1600-0757.2005.00108.x 15853935

[B18] Kononen E Development of oral bacterial flora in young children Ann Med 2000 32 107 112 10.3109/07853890009011759 10766401

[B19] Kolenbrander PE Oral microbial communities: Biofilms, interactions, and genetic systems Annu Rev Microbiol 2000 54 413 437 10.1146/annurev.micro.54.1.413 11018133

[B20] Preza D Olsen I Willumsen T Grinde B Paster B Diversity and site-specificity of the oral microflora in the elderly Eur J Clin Microbiol Infect Dis 2009 28 1033 1040 10.1007/s10096-009-0743-3 19373498 PMC2821189

[B21] Nyvad B Microbial colonization of human tooth surfaces APMIS Suppl 1993 32 1 45 8494649

[B22] Kilian M Reinholdt J Lomholt H Poulsen K Frandsen EV Biological significance of IgA1 proteases in bacterial colonization and pathogenesis: critical evaluation of experimental evidence APMIS 1996 104 321 338 10.1111/j.1699-0463.1996.tb00724.x 8703438

[B23] Mager DL Ximenez-Fyvie LA Haffajee AD Socransky SS Distribution of selected bacterial species on intraoral surfaces J Clin Periodontol 2003 30 644 654 10.1034/j.1600-051X.2003.00376.x 12834503

[B24] Lie MA Timmerman MF Velden U van der Weijden GA van der Evaluation of 2 methods to assess gingival bleeding in smokers and non-smokers in natural and experimental gingivitis J Clin Periodontol 1998 25 695 700 10.1111/j.1600-051X.1998.tb02509.x 9763323

[B25] Barendregt DS Timmerman MF Velden U van der Weijden GA van der Comparison of the bleeding on marginal probing index and the Eastman interdental bleeding index as indicators of gingivitis J Clin Periodontol 2002 29 195 200 10.1034/j.1600-051x.2002.290302.x 11940136

[B26] Gerardu VAM Buijs MJ van Loveren C ten Cate JM Plaque formation and lactic acid production after the use of amine fluoride/stannous fluoride mouthrinse Eur J Oral Sci 2007 115 148 152 10.1111/j.1600-0722.2007.00436.x 17451506

[B27] Huse SM Dethlefsen L Huber JA Mark Welch D Relman DA Sogin ML Exploring microbial diversity and taxonomy using SSU rRNA hypervariable tag sequencing PLoS Genet 2008 4 e1000255 10.1371/journal.pgen.1000255 19023400 PMC2577301

[B28] Pruesse E Quast C Knittel K Fuchs BM Ludwig W Peplies J Glockner FO SILVA: a comprehensive online resource for quality checked and aligned ribosomal RNA sequence data compatible with ARB Nucl Acids Res 2007 35 7188 7196 10.1093/nar/gkm864 17947321 PMC2175337

[B29] Cole JR Chai B Farris RJ Wang Q Kulam SA McGarrell DM Garrity GM Tiedje JM The Ribosomal Database Project (RDP-II): sequences and tools for high-throughput rRNA analysis Nucl Acids Res 2005 33 D294 296 10.1093/nar/gki038 15608200 PMC539992

[B30] Schloss PD Handelsman J Introducing DOTUR, a computer program for defining operational taxonomic units and estimating species richness Appl Environ Microbiol 2005 71 1501 1506 10.1128/AEM.71.3.1501-1506.2005 15746353 PMC1065144

[B31] Hammer O Harper DAT Ryan PD PAST: Paleontological statistics software package for education and data analysis Palaeontologia Electronica 2001 4 1 9

